# Metagenomic views of microbial dynamics influenced by hydrocarbon seepage in sediments of the Gulf of Mexico

**DOI:** 10.1038/s41598-020-62840-z

**Published:** 2020-04-01

**Authors:** Rui Zhao, Zarath M. Summers, Glenn D. Christman, Kristin M. Yoshimura, Jennifer F. Biddle

**Affiliations:** 1 School of Marine Science and Policy, University of Delaware, Lewes, DE United States; 2 Corporate Strategic Research, ExxonMobil Research and Engineering, Annandale, NJ United States

**Keywords:** Ecology, Biogeochemistry, Ecology, Ocean sciences

## Abstract

Microbial cells in the seabed are thought to persist by slow population turnover rates and extremely low energy requirements. External stimulations such as seafloor hydrocarbon seeps have been demonstrated to significantly boost microbial growth; however, the microbial community response has not been fully understood. Here we report a comparative metagenomic study of microbial response to natural hydrocarbon seeps in the Gulf of Mexico. Subsurface sediments (10–15 cm below seafloor) were collected from five natural seep sites and two reference sites. The resulting metagenome sequencing datasets were analyzed with both gene-based and genome-based approaches. 16S rRNA gene-based analyses suggest that the seep samples are distinct from the references by both 16S rRNA fractional content and phylogeny, with the former dominated by ANME-1 archaea (~50% of total) and Desulfobacterales, and the latter dominated by the Deltaproteobacteria, Planctomycetes, and Chloroflexi phyla. Sulfate-reducing bacteria (SRB) are present in both types of samples, with higher relative abundances in seep samples than the references. Genes for nitrogen fixation were predominantly found in the seep sites, whereas the reference sites showed a dominant signal for anaerobic ammonium oxidation (anammox). We recovered 49 metagenome-assembled genomes and assessed the microbial functional potentials in both types of samples. By this genome-based analysis, the seep samples were dominated by ANME-1 archaea and SRB, with the capacity for methane oxidation coupled to sulfate reduction, which is consistent with the 16S rRNA-gene based characterization. Although ANME-1 archaea and SRB are present in low relative abundances, genome bins from the reference sites are dominated by uncultured members of NC10 and anammox *Scalindua*, suggesting a prevalence of nitrogen transformations for energy in non-seep pelagic sediments. This study suggests that hydrocarbon seeps can greatly change the microbial community structure by stimulating nitrogen fixation, inherently shifting the nitrogen metabolism compared to those of the reference sediments.

## Introduction

Abundant microbial cells are found in marine sediments beneath the ocean, accounting for 1/3^rd^ to 1/20^th^ of total microbial biomass on the planet^1^. Microbial cells in sediments beyond the bioturbation zone are thought to experience extremely low energy availability^2^, and persist in a maintenance state without proliferation for at least tens of years^3^. At a sediment site with a near-constant sedimentation rate (i.e. under a steady state), microbial biomass follows a power-law decreasing trend with depth^1,4^, presumably due to the decrease of energy availability^2^. The responses of sedimentary microbial communities to environmental changes was documented in Peru Margin sediments, where cell increases were seen in relation to the sulfate-methane transition zones^5,6^. Subseafloor microbes were also observed to uptake amended substrates and proliferate in laboratory incubations^7,8^. However, their responses to other environmental disturbances and energy pulses have not been well studied.

Natural seafloor hydrocarbon fluid and gas seepage is a prevalent process on the continental margin^9^ and pelagic area^10^ of the Gulf of Mexico (GOM), and could serve as an important environmental influence on the local sedimentary microbial community. These seep fluids are enriched in carbon and can markedly alter the microbial community structure and function^11,12^. Metagenome sequencing has been previously performed to better assess the microbial response to petroleum seeps in GOM^13^. However, most of the analyses performed focused on functional genes^14,15^ rather than genomes. Microbial genomes in hydrocarbon-impacted sediments were reported in the Guaymas Basin^16^ and compared to the nearby reference sites^17^, to elucidate the changes in metabolic functionality and dependencies caused by hydrocarbon seepages. In Guaymas Basin sediments, despite contrasting community compositions, similar overall community functionality was observed between hydrothermal and non-hydrothermal sediments^17^. Functional redundancy of the microbial communities was invoked to explain this observation^17^.

In this study, we collected sediment cores from multiple sites with and without natural hydrocarbon seepages in the GOM to investigate the impacts of this environmental disturbance to microbial communities. We focus on the subseafloor sediments to test the hypothesis that the seep-related communities persist at low metabolic rates in the deep sediments and are insensitive to hydrocarbon seepages, as had been previously suggested for GOM sediments^11^. Here we employ both gene- and genome-based approaches to characterize the microbial community composition and structure, overall metabolic functionality, and inter-dependencies by comparing the metagenomes from seep and reference environments. With our genomic and functional insights into the microbial communities inhabiting the two contrasting geochemical conditions, we see that in addition to microbial cycling of sulfate and methane, nitrogen cycling dynamics must be severely shifted between seep and reference sediments.

## Materials and methods

### Sampling and site characterization

**Figure 1:**
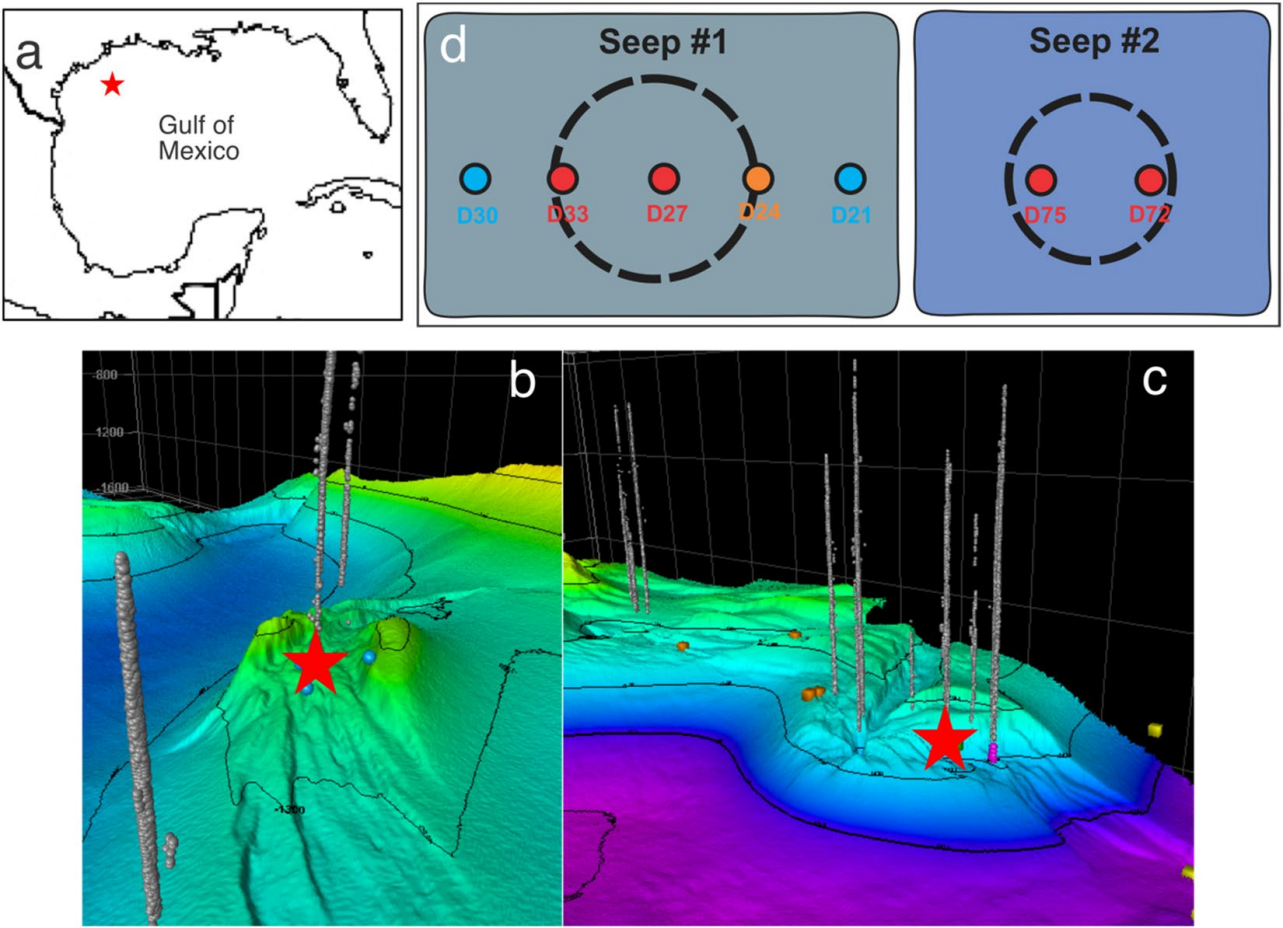
Sampling locations of this study. (**a**) Map of the sampling area in the Gulf of Mexico (GOM) prepared using R package *maps* (https://cran.r-project.org/web/packages/maps/index.html). **(b**,**c)** Bathymetric map of Seep-1 (**b**) and Seep-2 (**c**) in GOM. Natural seepage detected by sonar is indicated by the grey gas bubbles. Images were created using QPS Fledermaus software (https://www.qps.nl/fledermaus/) (**d**) Settings of the 7 sampling sites: seep sites are shown in red circles and reference sites without seepage influence are shown in blue circles. D24 (marked in orange) was retrieved from the edge of Seep-1, but no geochemical signal of seepage was detected.

Samples used in this study were retrieved by multiple push coring via a remotely operated vehicle aided by video feed, from two contrasting areas: Seep-1 and Seep-2 in the seabed of the Gulf of Mexico (GOM) (Table 1; Fig. 1a). Seeps were identified using a hull mounted multibeam echosounder which identified the natural gas plumes as indicators of active seepage as well as the nature of the seafloor (Fig. 1b,c). Sediment cores were retrieved by an ROV using a push-corer. In Seep-1, one core (D27) was collected from the center, two cores (D21 and D30) were collected from the edge, and the other two samples (D24 and D33) were collected halfway between the center and edge of this seep location (Fig. 1d). In Seep-2, two cores were retrieved from the central area: D72 was a site with white bacterial mat, while D75 was a site where seep bubbles were observed. Sediments of 10–15 cm below seafloor (cmbsf) of each core were sterilely subsampled, stored in an Falcon tube, and preserved in liquid nitrogen for transport to an onshore laboratory where they were stored at −80 °C until further DNA-based analyses were performed.

**Table 1:** Samples and metagenome data used in this study.

Sample ID	Sample information^a^	Category	DNA concentration (ng/uL)	DNA yield (ng/g)	Total basepair (Gbp^b^)	#Contigs
D21	100 m outside Seep-1	Reference	1.09	218	10.9	833,842
D24	½ way between the center to outside of Seep-1	Transition	1.11	222	10.7	644,534
D27	Center of Seep-1	Seep	4.95	990	16.5	659,874
D30	100 m outside Seep-1(opposite direction from D21)	Reference	1.20	240	9.8	505,334
D33	Edge of Seep-1	Seep	6.63	1326	17.7	652,345
D72	Seep site of Seep-2, oily bubbles observed	Seep	8.26	1652	17.0	602,628
D75	Bacterial mat of Seep-2	Seep	7.65	1530	16.1	586,157

^a^all samples were collected from 10–15 cm below seafloor.

^b^Gigabasepairs.

Each push core collected was subsampled for a variety of analyses. After sub-sampling all remaining sediments were placed in 500 ml isojars and poisoned with zephiran chloride. Geochemistry measurements were performed at Isotech Laboratories (Champagne, IL, USA). Liquid hydrocarbon presence/absence was determined for all push core samples (seep and background) based on maximum florescence intensity (MFI) and unresolved complex mixture (UCM) as screening tools, and detailed GC chromatograms for confirmation. Dissolved hydrocarbon concentrations were determined in seawater above the active seep bubble plumes at site D27. Sample was taken 1 meter above origin of the visible bubble plumes and analyzed by Isotech Laboratories (Champagne, IL, USA) for hydrocarbon composition and composition of methane, ethane, and propane.

### DNA extraction and metagenomic sequencing

For each sample, DNA was extracted from 0.5 gram (duplicate extractions with 0.25 gram used for each) of sediment using the MoBio PowerSoil DNA isolation kit following the manufacturer’s instructions. Resulting DNA from the two duplicate extractions were pooled and eluted into 100 uL of PCR grade water. DNA concentrations were measured using a Qubit 2.0 fluorometer (Thermo Fisher Scientific). Metagenomic libraries were prepared using NEBNext UltraN II DNA library preparation kit and sequenced (2 × 250 bp) on an Illumina HiSeq platform (Illumina Inc.) at the Sequencing & Genotyping Center, University of Delaware.

### Quantitative PCR

The bacterial 16S rRNA gene was quantified using the primer pair B1114/B1275R and thermal cycling conditions as described in Yoshimura, *et al*.^18^. Archaeal 16S rRNA gene was quantified using primers Uni515F/ Arc908r and thermal cycling conditions as described in Zhao, *et al*.^19^. The *mcrA* gene, encoding the alpha subunit of Methyl-CoM reductase (MCR) complex, was quantified using the primers qmcrA-F/qmcrfA-R^20^, combining with the thermal cycling conditions previously described^18^. All qPCR reactions were run in triplicate and each reaction mixture contained 1 × QuantiTech SybrGreen PCR master mixture (QIAgen, Germany), 0.5 μM forward and reverse primer and 1 μl of DNA template in a final volume of 20 μL. The standard for each gene was linear genomic DNA from *E. coli* (bacterial 16S rRNA gene) or *Methanosarcina mazei* (archaeal 16S rRNA gene and *mcrA* gene). For each gene, the DNA concentration of the standard was measured using Qubit and a DNA abundance gradient of 10–10^8^ copies µL^−1^ was prepared by 10x serial dilution. Gene abundances were normalized to copies per gram sediment.

### Metagenome data analysis

Raw metagenome sequencing reads were first quality controlled using FastQC v0.11.5^21^, and then trimmed using Trimmomatic v0.36^22^ to trim read-through adapters (ILLUMINACLIP:TruSeq. 2-PE.fasta:2:30:10), trim low quality base calls at the starts and ends of reads (LEADING:3, TRAILLING:3), remove reads that had average phred score lower than 20 in a sliding window of 10 bp (SLIDINWINDOW:10:20), and finally remove reads shorter than 100 bp (MINLEN:100). The overall quality of processed reads was evaluated again with FastQC v0.11.5, to ensure only high-quality reads were used in the downstream analysis.

### Gene-based analysis

Short reads of the 16S rRNA gene in the trimmed sequences were identified and taxonomically classified using two different programs: phyloFlash v3.2 beta1^23^ and GraftM v0.12.2^24^. The former program uses BBMap^25^ to identify and VSEARCH v2.5.0^26^ to classify putative 16S rRNA gene reads with SIVLA 132 release^27^ as the reference database, while the latter program uses curated packages prepared from RDP^28^ to identify 16S rRNA gene reads and pplacer^15^ to assign the taxonomy.

In addition, putative functional genes encoding key enzymes in nitrogen, sulfur, and methane cycling pathways were also identified and classified using GraftM^24^ with the default parameters and the respective curated reference packages (available at https://data.ace.uq.edu.au/public/graftm/7/). The relative abundances of each gene in each sample were normalized by dividing by the total read pairs in the trimmed dataset of that sample.

### Metagenome assembly and genome binning

Metagenome sequencing data from the samples of the two different groups (Seep sites: D27, D33, D72 and D75; Reference sites: D21 and D30) were processed separately; the transition site, D24, was not analyzed further. To maximize assembly, reads from different samples of the same group were co-assembled using megahit v2^29^ with the following parameters:–k-min 21,–k-max 121,–k-step 10,–min-contig-len 500. Contigs longer than 1 kb were subjected to automatic genome binning with MaxBin v2.2.5^30^ using the maximum expectation algorithm based on the tetranucleotide frequency, %GC, and differential coverage. The quality of the resulting genome bins were evaluated with CheckM^31^ using the default parameters. Genome bins of contamination level >10% were manually refined using gbtools^32^, taking the %GC, marker gene taxonomy, and differential coverages in different samples into account. The qualities of the final bins were quality checked using CheckM again, only those of >50% of completeness and <10% of contamination were retained as metagenome-assembled genomes (MAGs) and reported in this study, to include most of the final MAGs to discuss the overall metabolic shift of sedimentary microbial communities caused by hydrocarbon emission. To determine the relative abundance of each MAG in each sample, coverage of each genome was determined by mapping the trimmed reads onto the genome assembly using BBmap v37.61^25^, with the similarity threshold of 98%. Coverages were processed in R^33^ and visualized by heatmaps prepared using the R package *ggplot2*^34^.

### Genome annotation and functional characterization

Genome bins were annotated using Prokka v1.13.3^35^, with default settings for bacteria and the “–kingdom archaea” flag for archaeal genomes. Putative coding DNA sequences (CDS) were predicted by Prodigal^36^ in the Prokka annotation process. The functional assignments of genes of interest were also confirmed using BLASTp^37^ and annotations of KEGG^38^ and eggNOG^39^. The overall metabolic capacities of MAGs were visualized through a heatmap prepared using KEGG-decoder V1.0.6–1.0.8^40^ based on the annotation results from KEGG annotation.

### Phylogenetic analysis

Phylogenomic analysis was performed based on the concatenation of 13 ribosomal proteins (rpL2, 3, 4, 5, 6, 14, 15, 18, 22, 24 and rpS3, 8, 10, 17, 19). These ribosomal proteins have been demonstrated to undergo limited lateral gene transfer^41^, and have been included in large scale phylogenetic analysis of bacteria, archaea, and eukaryotes^42^. Close relative genomes of the MAGs recovered in this study were identified by BLASTp search^43^ in the NCBI webserver, using the ribosomal protein L2 as the query. These ribosomal proteins in each genome (the 49 MAGs recovered from GOM and their close relatives), among the conservative single-copy ribosomal proteins included in Campbell, *et al*.^44^, were identified in Anvi’o v5.4^45^ by Hidden Markov Model (HMM) search following the procedure described here (http://merenlab.org/2017/06/07/phylogenomics/). Sequences were aligned individually using MUSCLE^46^ and gaps were removed using trimAl^47^ with the “automated1” mode. Individual alignments were concatenated and the maximum likelihood phylogenetic tree was reconstructed using RAxML^48^ using PROTGAMMALG as the substitution model. Interactive tree of life (iTOL)^49^ was used for tree visualization and modification.

For the phylogenies of nitrogenases (NifH and NifK), the corresponding protein sequences from the GOM genomes were manually extracted from the Prokka annotation outputs. These protein sequences were also used as the queries in BLASTp^43^ searches in the NCBI webserver, to identify their close relatives (up to 5 close relatives were retained). For each of the two proteins, all sequences were aligned using MAFFT LINSi^50^ and gaps were removed using trimAl^47^ with the “automated1” mode. Maximum-likelihood phylogenetic trees were reconstructed using IQ-TREE v.1.6.10^51^ with substitution model determined by ModelFinder^52^, and 1000 ultrafast bootstrap replicates.

## Results

We collected 7 sediment cores from two seeps and their adjacent areas in GOM (Fig. 1, and Table 1). Thermogenic gases were detected in most of the subsurface sediments retrieved from the two seep sites (except D24), but not the reference sites. In addition, liquid hydrocarbons were also noted in the two sediments (D72 and D75) from Seep-2 (Table 2). At the site with visible bubble plume, D27 at the center of Seep-1 area, thermogenic gases were also detected in the overlying seawater (139 ppm methane, 0.16 ppm ethane, and 0.049 ppm of propane). Based on these geochemical characterizations, we divided these samples into two groups: D27, D33, D24, D72 and D75 are within seep areas influenced by natural liquid and gaseous hydrocarbon seepages, while D21 and D30 serve as references without obvious seepage influences.

**Table 2:** Seepage materials detected in sediment samples.

Sample	Thermogenic Gases Detected^a^	Liquid Hydrocarbons Detected^b^
D21	No	No
D30	No	No
D24	No	No
D27	Yes	No
D33	Yes	No
D72	Yes	Yes
D75	Yes	Yes

^a^Gas was measured by gas chromatography.

^b^Oil presence was determined based on maximum florescence intensity and unresolved complex mixture analyses.

### DNA concentrations and gene abundances

**Figure 2:**
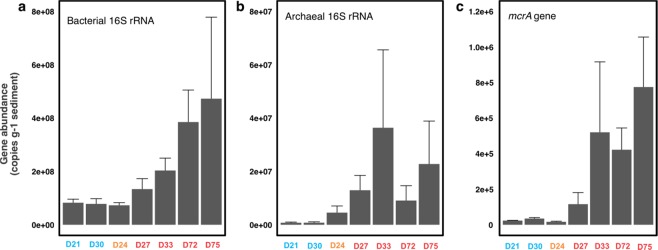
Abundances of bacterial (**a**) and archaeal (**b**) 16S rRNA genes and *mcrA* genes (**c**) in the GOM sediments. Seep sites were shown in red, while the reference sites were shown in blue. D24, shown in orange, was at the transition site. Error bars indicate the standard deviation of the triplicate qPCR measurements.

We extracted the total DNA and used qPCR to quantify the abundance of both the archaeal and bacterial 16S rRNA genes. With the same input materials in DNA extraction, we obtained higher DNA yields in the seep sites than the reference sites (Student *t*-test, *t* = −7.92, *P* = 0.004) (Table 1). Consistent with this, bacterial 16S rRNA gene abundances were detected to be 1.3–4.7 × 10^8^ copies g^−1^ at the seep sites, while only 7.8–8.3 × 10^7^ copies g^−1^ were detected at the reference sites (Student *t*-test, *t* = −2.81, *P* = 0.067) (Fig. 2a). Likewise, archaeal 16S rRNA gene abundance was quantified to be 0.9–3.6 × 10^7^ copies g^−1^ at the seep sites, while only 7.6–7.8 × 10^5^ copies g^−1^ at the reference site (Student *t*-test, *t* = −2.94, *P* = 0.055) (Fig. 2b). Higher DNA yields and 16S rRNA gene abundances detected at the seep sites indicate hydrocarbon seepage increases the overall microbial abundance.

### 16S rRNA gene analysis of metagenome

**Figure 3:**
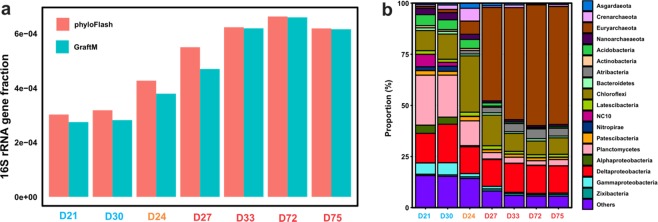
Fractions of 16S rRNA gene fragments (**a**) and community composition based 16S rRNA gene fragments detected in the unassembled metagenome reads (**b**). Comparative results were obtained from the two different programs (phyloFlash in red, and graftM in blue). The 16S rRNA reads were identified by phyloFlash v3.2 beta1^23^ and classified using VSEARCH v2.5.0^26^ against the SILVA 132 reference database. In both (**a**,**b**), samples are color coded as outside seep (blue), transition (orange) and inside seep (red).

Illumina sequencing of the metagenomic libraries from the GOM sediments produced 9.8–17.0 Gigabasepair (Gbp) of quality-filtered reads in individual samples (Table 1). Using both GraftM and phyloFlash, we detected higher proportions of short 16S rRNA gene reads in the seep sites (0.047–0.066%) relative to the reference sites (0.027–0.038%) (Student *t*-test, *t* = −5.21, *P* = 0.0034) (Fig. 3a). Intermediate fractions were observed in the site D24.

Different community structures were observed based on 16S rRNA genes at the two types of sediment sites, although the community compositions were similar (Fig. 3b). Microbial communities at the seep sites were dominated by archaea, especially the ANME-1 archaea within the phylum of Euryarchaeota (45.8–59.3% of total community), which is in agreement with the fact that these are active seep sites, supported by the visible methane gas plumes present at both sites (Fig. 1b,c) and detection of gas (Table 2). Other dominant taxa (Euryarchaeota, Chloroflexi, Deltaproteobacteria, and Atribacteria) present in the seep samples were also detected in the reference sites (Fig. 3b). In contrast, the reference sites were dominated by bacteria, especially members of Chloroflexi, Planctomycetes (mainly the genus of *Scalindua*), and Deltaproteobacteria, with archaea only accounting for small fractions (8.8–11.0% of the total community) (Fig. 3b). We note that Deltaproteobacteria and, to a lesser extent (<2%), Latescibacteria, Patescibacteria (i.e. Candidate Phyla Radiation, CPR) and Zixibacteria were present with similar proportions in the two types of sediments.

### Functional gene fractions

**Figure 4:**
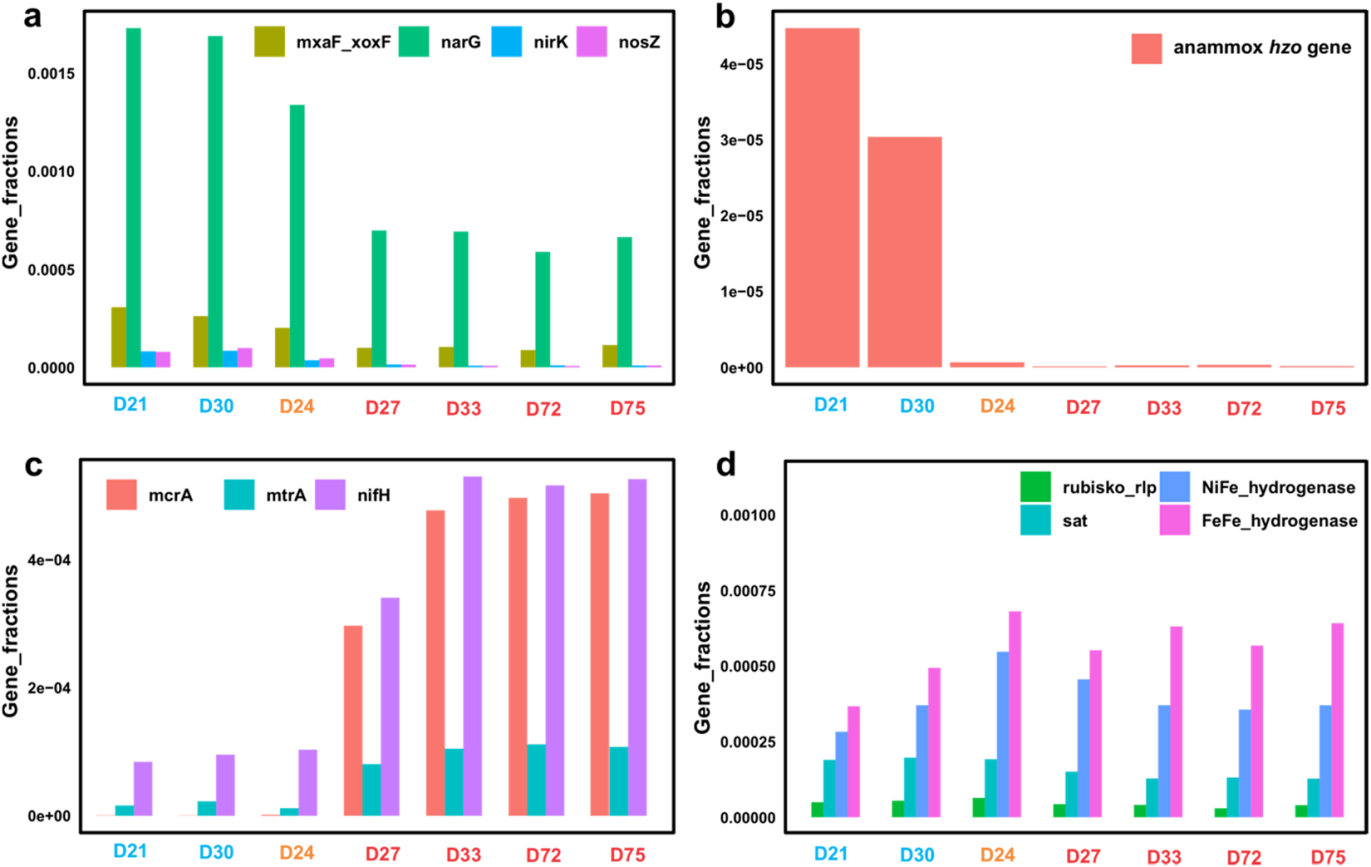
Fractions of functional genes in the unassembled metagenome reads. (**a**) Relative abundance of key genes of denitrification (*narG*, nitrate reductase; *nirK*, nitrite reductase; *nosZ* nitrous oxide reductase) and aerobic methane oxidation (*mxaF*/*xoxF*). (**b**) Relative abundance of genes for annamox (*hzo*, hydrazine dehydrogenase). (**c**) Fractions of genes for anaerobic methane generation and oxidation (*mcrA* and *mtrA*), nitrogen fixation (*nifH*). (**d**) Fractions of genes of carbon fixation (RuBisCo), sulfate reduction (sat) and hydrogen oxidation (hydrogenase). Samples are color coded as in Fig. 2.

We used GraftM to detect various functional genes involved in biogeochemical cycles. We detected higher fractions (0.03–0.05%) of *mcrA* gene encoding the methyl coenzyme M reductase involved in methane/alkane metabolism at the seep sites, while *mcrA* is virtually absent in the reference sites (Fig. 4c). This observation was also confirmed by *mcrA* gene-specific qPCR, in which ~20-fold higher *mcrA* gene abundances were quantified in the seep sites than the reference sites (Fig. 1c). In addition, the *nifH* gene which encodes nitrogenase, the key enzyme of nitrogen fixation process, was also observed to be higher in the seep sites than the reference sites (Student *t*-test, *t* = −8.30, *P* = 0.0033) (Fig. 4c). The majority of the gene fragments of these two genes (92–95% of *mcrA* and 32–39% of *nifH*) were contributed by contributed to by genes that are similar to those from known euryarchaeotal organisms.

We observed higher fractions of functional genes for denitrification (*narG*, *nirK*, and *nosZ*) at the reference sites than the seep sites (Fig. 4a). Similarly, *mxaF*_*xoxF* gene encoding the alpha subunit of methanol dehydrogenase in aerobic methane oxidizing bacteria^53^, is also observed to show higher percentages in the reference sites than the seep sites (Fig. 4a). These results suggest that the reference sediments harbor more microbes capable of utilizing nitrate and oxygen as electron acceptors in redox reactions. In addition, the functional gene for anaerobic ammonium oxidation (*hzo* encodes the hydrazine dehydrogenase of the last step of anammox) was mostly detected in the two reference sites (Fig. 4b), suggesting that anammox may only be significant in the reference sites. In contrast, genes of sulfate reduction (*aprA* encoding adenosine-5-phosphosulfate (APR) reductase subunit alpha and *sat* encoding sulfate adenylyltransferase), carbon fixation (RuBisCo), and hydrogen utilization (NiFe- and FeFe-hydrogenase) were also detected in both groups of samples, without significant differences in the relative abundances between the two groups of samples (Fig. 4d).

### MAG distribution and function in GOM sediments

**Figure 5:**
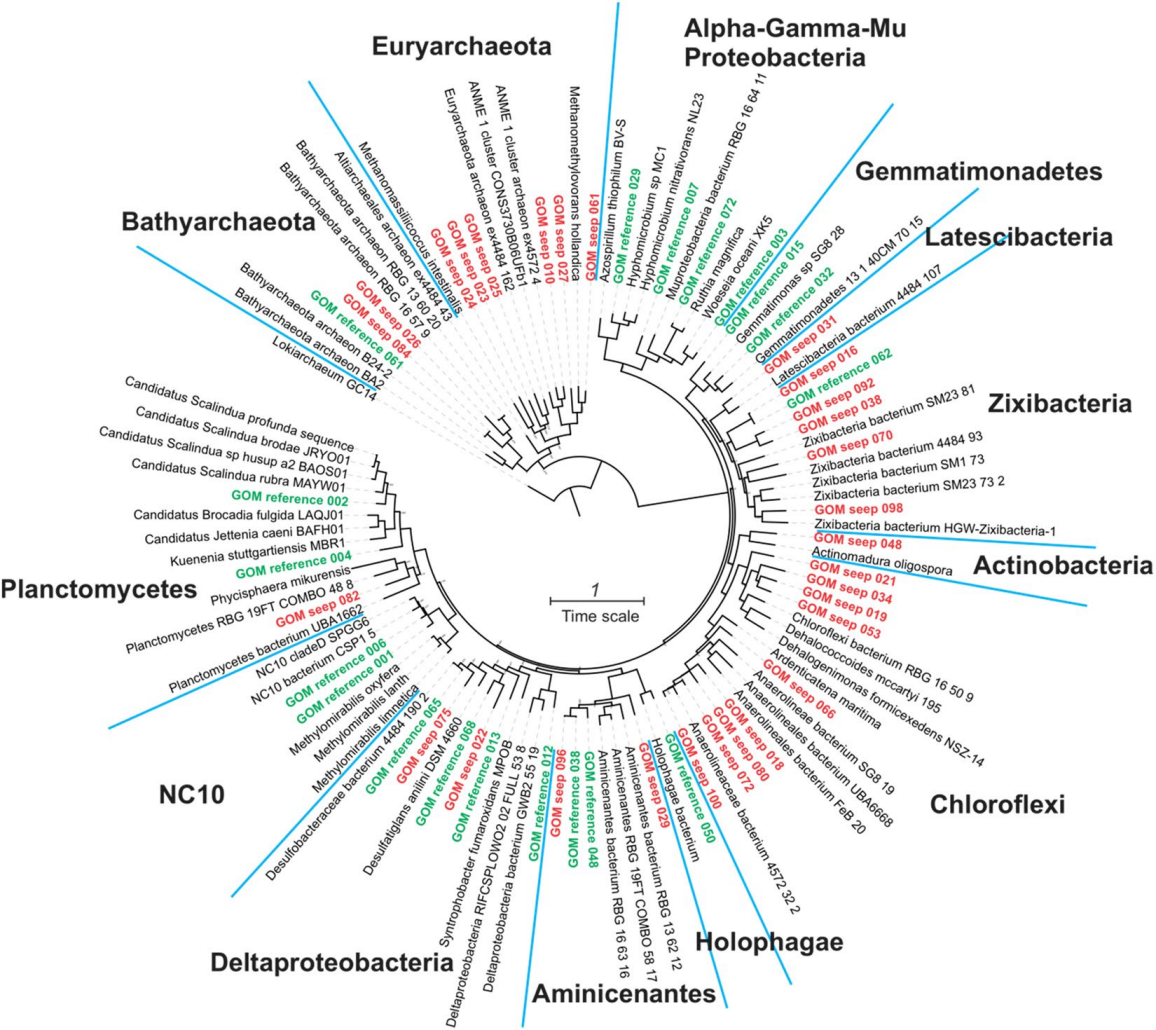
Maximum-likelihood phylogenetic tree of MAGs. The tree was reconstructed using RAxML v8.2.8^48^ with the PROTGAMMALG model, based on the alignment of 13 concatenated ribosomal proteins (rpL2, 3, 4, 5, 6, 14, 15, 18, 22 and rpS8, 10, 17, 19). The tree is rooted to *Canditatus* Lokiarchaeota GC14–75. MAGs from the GOM seep sites are highlighted in red, and MAGs from the reference sites are marked in green. Scale bar represents 1 amino acid substitution per sequence position.

**Figure 6:**
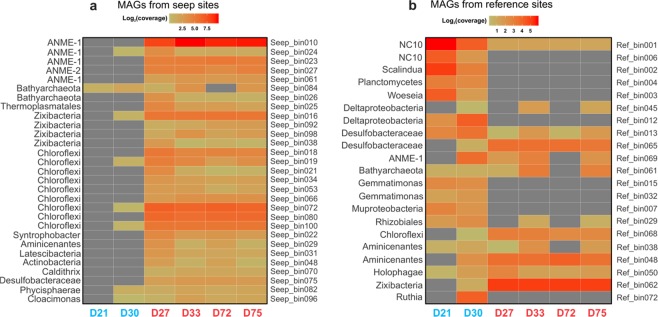
Coverage of genome bins recovered from (**a**) the seep and (**b**) reference sites. Genome coverages (used as a proxy of the relative abundance of MAGs in complex commuties) were determined by read mapping using BBMap v37.61^25^ and processed in R^33^ and visualized by heatmaps prepared using the R package *ggplot2*^34^.

Following metagenome assembly and binning, a total of 49 MAGs (29 from the seep samples and 20 from the reference samples) were recovered from the metagenome datasets, excluding sample D24. Each MAG was classified by constructing a phylogenetic tree using concatenated 13 single-copy ribosomal proteins (Fig. 5). These MAGs represent 13 bacterial and 2 archaeal phyla commonly found in marine sediments. The bacterial MAGs were dominated by the phyla of Chloroflexi (Anaerolineae and Dehalococcoidia), Deltaproteobacteria (Desulfobacteraceae and Syntrophobacter), Aminicenantes, NC10, Planctomycetes, and Zixibacteria (Fig. 6a). Archaeal MAGs was dominated by the phyla of Euryarchaeota (ANME-1 and ANME-2) and Bathyarchaeota (Fig. 6a). However, their distributions (using the average coverage of each MAG in each sample as the proxy) are markedly different between the sites with and without seepage influences (Fig. 6b). MAGs recovered from one environment are generally absent in the other environment (Fig. 6), except for the MAGs of Bathyarchaeota and Deltaproteobacteria, which seemed to be cosmopolitan in both environments as suggested by the 16S rRNA gene analysis (Fig. 2b).

Consistent with the 16S rRNA gene analysis, MAGs recovered from the seep sites were dominated by Euryarchaeota (mainly ANME-1 archaea), Bathyarchaeota, Anaerolineae, Aminicenantes, and Zixibacteria (Fig. 6b). Some ANME archaeal MAGs from the seep sites, including GOM_Seep_010, GOM_Seep_023, and GOM_Seep_027, encode molybdenum-dependent nitrogenase (Nif) that could enable them to fix dinitrogen gas to ammonia (Fig. S1). In contrast, MAGs from the reference sites without seepage influences were dominated by bacteria from the taxa of NC10, *Scalindua*, *Woeseia*, Deltaproteobacteria, and Gemmatimonas (Fig. 6b). We also found a novel bacterial MAG affiliated to the recently proposed phylum Muproteobacteria, which is absent in the seep sites (Fig. 5).

## Discussion

We performed a metagenomic study focusing on subseafloor sediments from two contrasting habitats in the Gulf of Mexico to examine the microbial responses to seafloor hydrocarbon seepage. We used five samples from sites bearing natural hydrocarbon seepage and two from nearby reference sites without detectable seepage. Based on gene- and genome-centric analyses, this study reveals that hydrocarbon seepage can significantly alter the community structure. Although metagenome-assembled genomes are helpful to provide information of microbes in natural environments beyond their identities^16,17,54,55^, complete microbial genomes are rarely reconstructed from natural environments^56^. Importantly, reconstructed genomes often lack 16S rRNA^57^, which is a robust phylogenetic marker and is critical to constrain the taxonomy of novel genomes on the basis of existing extensive 16S rRNA gene sequences^23^. Also, the assembly-binning approaches typically only recover members of a community that have sufficient coverage, minimal population heterogeneity, few sequence repeats and distinct genome nucleotide composition^58^. As a result, current genome-centric approaches do not capture the full phylogenetic and functional diversity of a microbial community, particularly in complex environments. These deficiencies have led to the emergence of gene-centric analysis pipelines such as phyloFlash^23^ and graftM^24^, which bypass the bottleneck of assembly of complex communities and can provide a comprehensive view of diversity of the 16S rRNA gene and other functional genes based on well-curated reference databases. Combining our gene and genomic analysis results, we offer detailed insights into the subsurface community.

Microbial community at site D24 showed mixed features of both seep and reference sites, although this sample was collected within Seep-1. Most of the analyzed microbial features such as the DNA yield, 16S rRNA gene and *mcrA* abundances assessed by qPCR (Fig. 2), ratio of 16S rRNA gene in the metagenome (Fig. 3a), abundance of ANME archaea (Fig. 3b), and functional gene ratios of hydrogenase and nitrate reductase (Fig. 4a), was lower than the seep sites but higher than the two reference sites. Considering that hydrocarbon seepages in GOM are ephemeral and can migrate over time^59^, it is very likely that the duration time of seepage at this site was shorter than the rest seep sites. Therefore, we excluded this site for the MAG comparison analysis.

### High fractions of ribosomal RNA in the seep sites suggesting active growth

We detected significantly higher fractions of the 16S rRNA gene in the metagenome at the seep sites than the reference sites. Microbes with different growth rates tend to have different copies of ribosomal RNA operons (*rrn*), and microbes exhibiting higher reproductive rates were revealed to have higher *rrn* copy numbers^60^. Therefore, the copy number of genes encoding the ribosome has been suggested as a predictor of both growth rate and carbon use efficiency because of a proteome allocation trade-off^60^. The different overall microbial community structures, especially the markedly different proportions of archaea observed between the two types of sites, could explain the different fractions of 16S rRNA gene in the total communities. However, archaea are known to have fewer copies of *rrn* per genome than bacteria^61^ and therefore are not likely to cause the higher fractions of 16S rRNA genes detected in the metagenomes of the seep sites. The faster proliferation rates of microbes stimulated by energy-rich substances emitting from the subsurface seepages is the most plausible explanation of the higher fractions of *rrn* in the metagenomes of the seep sites. Faster growth rates at the seep sites could also explain the higher microbial abundances detected by qPCR measurements (Fig. 1). Microorganisms in marine sediments beyond the bioturbation zone are always assumed to persistent in a maintenance state with turnover time of hundreds of years;^62,63^ our data, however, reveal that subseafloor microbes can respond to and experience significant growth due to the impact of hydrocarbon seepages. Whether the subseafloor microbes are still undergoing growth or not is unclear. Experimental approaches such as H_2_^18^O-labeling^64,65^ are necessary to apply to marine sediments to confirm the proposed microbial *in situ* growth here and elsewhere^19^.

### The overall community structure and functionality differences between the two types of sites

The overall microbial community structure was significantly changed by the presence of active hydrocarbon seepage (Table 2; Fig. 3). The notable dominance of ANME archaea detected at the seep sites (~50% of total) can be explained by the growth stimulated by methane in hydrocarbon seepage. It is worth noting that such archaeal dominances were not detected in the seep sites by qPCR of 16S rRNA genes, which is likely attributed to the known primer biases against archaea^66,67^. In the reference sites Planctomycetes (mainly anammox *Scalindua*), NC10, Acidobacteria, Alphaproteobacteria and Gammaproteobacteria were more frequently detected, suggesting that these groups are well adapted to the typical pelagic sediment environment found in the reference sites^68^. The relative abundances of Deltaproteobacteria, Latescibacteria, and Zixibacteria are similar between the two types of sediments, indicating these are part of the core microbiome in the sediments and are not affected by the hydrocarbon seepage.

We also assessed the potential functional differences between the two types of environments based on the functional gene contents in the metagenomic datasets. We detected notably high fractions of *mcrA* and *nifH* genes in the seep sediment metagenomes (Fig. 4c), both of which are largely derived from ANME archaea^69,70^. ANME archaea were >45% of the taxa detected in the seep sites while virtually absent in the reference sites (Fig. 3). ANME archaea at the seep sites could have exhibited significant growth under the stimulation of hydrocarbon seepage, supported by the 20-fold higher *mcrA* gene abundances relative to the reference sediments (Fig. 2c). The nitrogen fixation capacity of ANME archaea could provide ammonia as the nitrogen source for biomass synthesis during the microbial proliferation in the seep sites. The bloom of ANME archaea have resulted in higher methane oxidation rates previously noted in GOM cold seep sediments compared to reference sediments^71^. The increase in the relative abundance of archaea was also observed in the hydrothermal sediments in Guaymas Basin^17^. We also detected significantly higher fractions of *mtrA* gene in the metagenome datasets at the seep sites, the function of which was suggested as iron oxidation^72^, suggesting that iron oxidation could be more strongly affected at the seep sites than the reference ones.

We detected notably higher fractions of the functional genes for denitrification (*narG*, *nirK* and *nosZ*), anammox (*hzo* gene), and aerobic methane oxidation (mxaF/xoxF gene) in the reference sites, suggesting that these processes are more prevalent in typical pelagic sediments. This is consistent with the fact that the genomes of *Woeseia* and NC10, both capable of nitrate/nitrite reduction, and anammox *Scalindua* were mainly or exclusively recovered in the reference sites by metagenome assembly and binning (Fig. 6). Interestingly, in the reference sites we also detected higher abundances of the *mxaF/xoxF* genes, which are involved in aerobic methane oxidation^53^. This suggests that methane consumption in the reference sites is largely coupled to oxygen respiration, and that oxygen may be more steadily available than in the seep sites. This increase in oxygen availability in the sediments is likely due to lower overall microbial activities than those in the seep sediments under the stimulation of reduced substance fluxes.

Functional genes of carbon fixation (RuBisCo), sulfate reduction (*sat* gene), and hydrogen oxidation (NiFe- and FeFe-hydrogenase) are not statistically different between the two types of sediments. This observation suggests that microorganisms responsible for these processes are core members of the microbial communities in these deep-sea sediments and are not affected by hydrocarbon seepages. This is consistent with the similar amounts of Deltaproteobacteria (mainly Desulfobacterales) detected in both environments by 16S rRNA gene-based analysis (Fig. 3b) since Desulfobacterales have been suggested to play an important role in H_2_ consumption and sulfate reduction in marine sediments^73^.

### Metabolic dependency between different groups in the two contrasting environments

**Figure 7:**
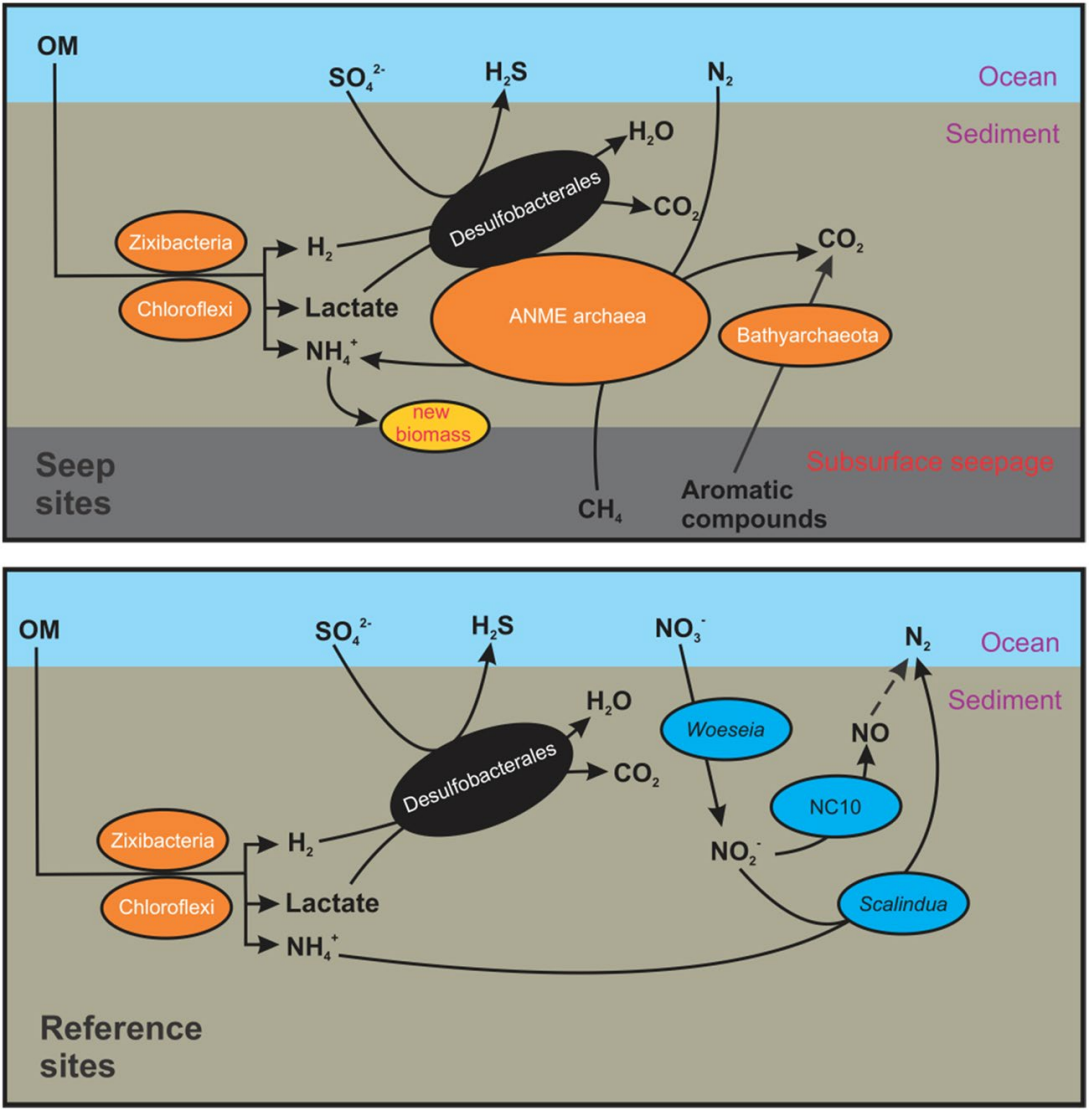
Major geochemical processes and the associated microbial genomes in the seep (upper) and reference (lower) sites of GOM. Only metabolic pathways of the dominant microbial genomes in the two contrasting sediment sites are shown. Arrows represent metabolic capabilities that were inferred in the MAGs reconstructed from GOM deep-sea sediments by genome annotation. Taxa enriched in the seep and reference sites are shown in orange and blue, respectively, while the common taxa in both sites are shown in black. OM: organic matter.

To better understand the biogeochemical impacts of hydrocarbon seepage on sedimentary microbial communities in the Gulf of Mexico, we examined the major ecological roles of the dominant genomes in the two contrasting types of sediments (Fig. 7). In the seep sites, Chloroflexi (mainly Anaerolineae) and Zixibacteria are the major organic matter degraders that are capable of degrading organic matter to low-molecular weight (LMW) carbon compounds such as acetate and ethanol^16,17,74^. These LMW compounds can be used as the electron donor by sulfate-reducing Desulfubacterales. In addition to using sulfate as the electron acceptor, Desulfubacterales may also form consortia with ANME archaea and accept electrons from ANME archaea when performing sulfate-dependent methane oxidation under the stimulation of the presumed upward flux of methane from the subsurface. In addition, the higher relative abundances of Bathyarchaeota in the seep sites (Fig. 6) may be due to their capability of aromatic compound degradation^75^ and could contribute to the hydrocarbon degradation in GOM seep sediments. The co-enrichment of ANME and Bathyarchaeota has also been reported in the hydrothermal-influenced biofilm on the chimney wall of the Soria Moria vent field^76^, supporting that these two archaeal groups may have similar responses to environmental impacts.

The initial hydrolysis and fermentation of organic matter in the reference sediments is likely performed by the abundant taxa Chloroflexi and Zixibacteria (Fig. S2). The further degradation of organic matter could be carried out by putative denitrifying bacteria capable of using nitrate/nitrite as the electron acceptors, such as *Woeseia*^77^ and NC10 bacteria^78^. Nitrite produced from nitrate reduction and ammonium produced from the degradation of organic matter could be consumed by microbes of the genus of *Scalindua* (Fig. 7), a specific taxon capable of anaerobic ammonium oxidation^79^. The dominance of these taxa in the recovered MAGs is consistent with higher fractions of denitrification and anammox functional genes detected in the reference sites, suggesting that nitrogen transformation for energy generation is prevalent in the typical pelagic sediments in GOM.

Our results run counter to conclusions from the previously studied seeps in the Gulf of Mexico, where deeper communities were not seen to be greatly impacted compared to the surface-expression of the seeps^11^. Perhaps the removal of the surface comparison allows a more even comparison between deep sediments. However, our study also runs counter to the data from Guaymas Basin sedimentary seeps, where communities in-seep and outside-of-seep were found to be functionally redundant^17^. Guaymas Basin sediments may be under different nutrient limitations compared to Gulf of Mexico sediments, so nutrient influences on microbial communities may not be as readily seen there. Our work shows that seepage influences on the microbial community structure should be complexed with additional measurements to fully understand what limits or supports community development and geochemical cycling.

Overall, the finding that nitrogen fixation is largely present at the seeps versus nitrogen loss (via denitrification and annamox) present at the reference sites (Fig. 4b), suggests that the carbon use efficiency varies drastically between the two settings. While carbon and nitrogen dynamics are not often focused in the seafloor, the study of soils has shown that C and N balances greatly impact microbial processing^80^ and nitrogen fixation has been documented in carbon enriched sites^12^. This contrasting nitrogen metabolism pattern in GOM sediments - the prevalence of nitrogen fixation at seep sites and nitrogen loss at non-seep sites - was also supported by ^15^N isotope compositions of previous studies^71^. Microbial selection toward nitrogen-fixing bacteria in marine sediments has also been reported in laboratory microcosm containing hydrocarbon (crude-oil)^81^. Considering GOM seep sediments are considered organic-rich (3–6% weight percent^71^) and nitrogen-poor (C/N ratio in the range of 20–40 in sediments <10 cm^71^), our results indicate that the newly fixed nitrogen by diazotrophs is a prerequisite of the rampant microbial growth in seep sediment since N limitation may limit microbial growth. Meanwhile, our results show that outside seep environments, the deep pelagic sediments (>1,400 m water depth) may use nitrogenous compounds as an energy source. This investigation of seep and reference sediments from the Gulf of Mexico deep biosphere shows that sedimentary microbes may be limited by different factors in adjacent settings due to the bottom-up environmental perturbation of natural hydrocarbon seepage.

## Conclusion

Our study revealed the responses of subseafloor microbial communities to hydrocarbon seepage in deepwater GOM, by employing both gene- and genome-based analysis of metagenomes. Particularly, based on 16S rRNA gene analysis, community structure was revealed to be significantly altered, with higher fractions of 16S rRNA gene and the notable dominance of ANME archaea in the seep sites. Further, functional gene analysis suggested that anaerobic methane oxidation and nitrogen fixation, both presumed to be performed by ANME archaea, were intensified by hydrocarbon seepage, while nitrogen transformation such denitrification and anammox are dominant in the reference sediments. These metabolic features predicted in gene-based analysis were confirmed by genomic analysis, with high relative abundances of ANME archaea in the seep sites and the dominance of denitrifying *Woeseia* and anammox *Scalindua* in the reference sites. Collectively, this study revealed that natural seafloor hydrocarbon seepages in the deep ocean can alter the microbial community structure and change the dominant redox regime from nitrogen loss for energy production to sulfate-based methane oxidation and nitrogen fixation.

## Supplementary information

Supplementary Information.

## Data Availability

Metagenomic sequencing data used in this study are available in NCBI under the BioProject number PRJNA553005.

## References

[1] Kallmeyer J, Pockalny R, Adhikari RR, Smith DC, D’Hondt S (2012). Global distribution of microbial abundance and biomass in subseafloor sediment. Proceedings of the National Academy of Sciences of the United States of America.

[2] Jørgensen, B. B. & Marshall, I. P. G. In Annual Review of Marine Science, Vol 8 Vol. 8 Annual Review of Marine Science (eds. Carlson, C. A. & Giovannoni, S. J.) 311–332 (2016).

[3] Braun S (2017). Microbial turnover times in the deep seabed studied by amino acid racemization modelling. Scientific Reports.

[4] Parkes RJ, Cragg BA, Wellsbury P (2000). Recent studies on bacterial populations and processes in subseafloor sediments: A review. Hydrogeology Journal.

[5] Contreras S (2013). Cyclic 100-ka (glacial-interglacial) migration of subseafloor redox zonation on the Peruvian shelf. Proceedings of the National Academy of Sciences.

[6] Parkes RJ (2005). Deep sub-seafloor prokaryotes stimulated at interfaces over geological time. Nature.

[7] Morono Y (2011). Carbon and nitrogen assimilation in deep subseafloor microbial cells. Proceedings of the National Academy of Sciences of the United States of America.

[8] Trembath-Reichert E (2017). Methyl-compound use and slow growth characterize microbial life in 2-km-deep subseafloor coal and shale beds. Proceedings of the National Academy of Sciences of the United States of America.

[9] Garcia-Pineda O (2016). Transience and persistence of natural hydrocarbon seepage in Mississippi Canyon, Gulf of Mexico. Deep-Sea Research Part Ii-Topical Studies in Oceanography.

[10] Chakraborty A (2018). Thermophilic endospores associated with migrated thermogenic hydrocarbons in deep Gulf of Mexico marine sediments. ISME Journal.

[11] Lloyd KG (2010). Spatial structure and activity of sedimentary microbial communities underlying a Beggiatoa spp. mat in a Gulf of Mexico hydrocarbon seep. PLoS One.

[12] Dekas AE (2018). Widespread nitrogen fixation in sediments from diverse deep-sea sites of elevated carbon loading. Environmental microbiology.

[13] Dong X (2019). Metabolic potential of uncultured bacteria and archaea associated with petroleum seepage in deep-sea sediments. Nature communications.

[14] Scott NM (2014). The microbial nitrogen cycling potential is impacted by polyaromatic hydrocarbon pollution of marine sediments. Frontiers in Microbiology.

[15] Mason OU (2014). Metagenomics reveals sediment microbial community response to Deepwater Horizon oil spill. ISME Journal.

[16] Dombrowski N, Seitz KW, Teske AP, Baker BJ (2017). Genomic insights into potential interdependencies in microbial hydrocarbon and nutrient cycling in hydrothermal sediments. Microbiome.

[17] Dombrowski N, Teske AP, Baker BJ (2018). Expansive microbial metabolic versatility and biodiversity in dynamic Guaymas Basin hydrothermal sediments. *Nature*. Communications.

[18] Yoshimura KM, York J, Biddle JF (2018). Impacts of salinity and oxygen on particle-associated microbial communities in the Broadkill River, Lewes DE. Frontiers in Marine Science.

[19] Zhao R, Hannisdal B, Mogollon JM, Jørgensen SL (2019). Nitrifier abundance and diversity peak at deep redox transition zones. Scientific Reports.

[20] Denman SE, Tomkins N, McSweeney CS (2007). Quantitation and diversity analysis of ruminal methanogenic populations in response to the antimethanogenic compound bromochloromethane. FEMS Microbiol. Ecol..

[21] Andrews, S. FastQC: a quality control tool for high throughput sequence data. https://www.bioinformatics.babraham.ac.uk/projects/fastqc/ (2010).

[22] Bolger AM, Lohse M, Usadel B (2014). Trimmomatic: a flexible trimmer for Illumina sequence data. Bioinformatics.

[23] Gruber-Vodicka, H. R., Seah, B. K. & Pruesse, E. phyloFlash — Rapid SSU rRNA profiling and targeted assembly from metagenomes. bioRxiv, 521922, 10.1101/521922 (2019). 10.1128/mSystems.00920-20 PMC7593591 33109753

[24] Boyd JA, Woodcroft BJ, Tyson GW (2018). GraftM: a tool for scalable, phylogenetically informed classification of genes within metagenomes. Nucleic Acids Research.

[25] Bushnell, B. BBMap: a fast, accurate, splice-aware aligner. (Ernest Orlando Lawrence Berkeley National Laboratory, Berkeley, CA (US), 2014).

[26] Rognes T, Flouri T, Nichols B, Quince C, Mahe F (2016). VSEARCH: a versatile open source tool for metagenomics. PeerJ.

[27] Quast C (2013). The SILVA ribosomal RNA gene database project: improved data processing and web-based tools. Nucleic Acids Research.

[28] Cole JR (2005). The Ribosomal Database Project (RDP-II): sequences and tools for high-throughput rRNA analysis. Nucleic acids research.

[29] Li DH, Liu CM, Luo RB, Sadakane K, Lam TW (2015). MEGAHIT: an ultra-fast single-node solution for large and complex metagenomics assembly via succinct de Bruijn graph. Bioinformatics.

[30] Wu YW, Simmons BA, Singer SW (2016). MaxBin 2.0: an automated binning algorithm to recover genomes from multiple metagenomic datasets. Bioinformatics.

[31] Parks DH, Imelfort M, Skennerton CT, Hugenholtz P, Tyson GW (2015). CheckM: assessing the quality of microbial genomes recovered from isolates, single cells, and metagenomes. Genome Research.

[32] Seah BK, Gruber-Vodicka H (2015). R. gbtools: interactive visualization of metagenome bins in R. Frontiers in Microbiology.

[33] R Development Core Team. R: A language and environment for statistical computing. R foundation for statistical computing Vienna, Austria (2011).

[34] Wickham, H. ggplot2: elegant graphics for data analysis. (Springer, 2016).

[35] Seemann T (2014). Prokka: rapid prokaryotic genome annotation. Bioinformatics.

[36] Hyatt D (2010). Prodigal: prokaryotic gene recognition and translation initiation site identification. BMC Bioinformatics.

[37] Altschul SF (1997). Gapped BLAST and PSI-BLAST: a new generation of protein database search programs. Nucleic Acids Research.

[38] Kanehisa M, Sato Y, Morishima K (2016). BlastKOALA and GhostKOALA: KEGG Tools for Functional Characterization of Genome and Metagenome Sequences. Journal of Molecular Biology.

[39] Huerta-Cepas J (2016). eggNOG 4.5: a hierarchical orthology framework with improved functional annotations for eukaryotic, prokaryotic and viral sequences. Nucleic Acids Research.

[40] Graham E, Heidelberg J, Tully B (2018). Potential for primary productivity in a globally-distributed bacterial phototroph. The ISME journal.

[41] Sorek R (2007). Genome-wide experimental determination of barriers to horizontal gene transfer. Science.

[42] Hug LA (2016). A new view of the tree of life. Nature microbiology.

[43] Altschul SF, Gish W, Miller W, Myers EW, Lipman DJ (1990). Basic local alignment search tool. Journal of Molecular Biology.

[44] Campbell JH (2013). UGA is an additional glycine codon in uncultured SR1 bacteria from the human microbiota. Proceedings of the National Academy of Sciences of the United States of America.

[45] Eren AM (2015). Anvi’o: an advanced analysis and visualization platformfor ‘omics data. PeerJ.

[46] Edgar RC (2004). MUSCLE: multiple sequence alignment with high accuracy and high throughput. Nucleic Acids Research.

[47] Capella-Gutierrez S, Silla-Martinez JM, Gabaldon T (2009). trimAl: a tool for automated alignment trimming in large-scale phylogenetic analyses. Bioinformatics.

[48] Stamatakis A (2014). RAxML version 8: a tool for phylogenetic analysis and post-analysis of large phylogenies. Bioinformatics.

[49] Letunic I, Bork P (2016). Interactive tree of life (iTOL) v3: an online tool for the display and annotation of phylogenetic and other trees. Nucleic Acids Research.

[50] Katoh K, Standley DM (2013). MAFFT multiple sequence alignment software version 7: improvements in performance and usability. Molecular Biology and Evolution.

[51] Nguyen LT, Schmidt HA, von Haeseler A, Minh BQ (2015). IQ-TREE: A Fast and Effective Stochastic Algorithm for Estimating Maximum-Likelihood Phylogenies. Molecular Biology and Evolution.

[52] Kalyaanamoorthy S, Minh BQ, Wong TKF, von Haeseler A, Jermiin LS (2017). ModelFinder: fast model selection for accurate phylogenetic estimates. Nature Methods.

[53] Lau E, Fisher MC, Steudler PA, Cavanaugh CM (2013). The methanol dehydrogenase gene, mxaF, as a functional and phylogenetic marker for Proteobacterial methanotrophs in natural environments. PLoS One.

[54] Lazar CS, Baker BJ, Seitz KW, Teske AP (2017). Genomic reconstruction of multiple lineages of uncultured benthic archaea suggests distinct biogeochemical roles and ecological niches. ISME Journal.

[55] Hug LA (2016). Critical biogeochemical functions in the subsurface are associated with bacteria from new phyla and little studied lineages. Environmental Microbiology.

[56] Albertsen M (2013). Genome sequences of rare, uncultured bacteria obtained by differential coverage binning of multiple metagenomes. Nature Biotechnology.

[57] Parks DH (2017). Recovery of nearly 8,000 metagenome-assembled genomes substantially expands the tree of life. Nature Microbiology.

[58] Sczyrba A (2017). Critical sssessment of metagenome interpretation-a benchmark of metagenomics software. Nature Methods.

[59] Garcia-Pineda O (2016). Transience and persistence of natural hydrocarbon seepage in Mississippi Canyon, Gulf of Mexico. Deep Sea Research Part II: Topical Studies in Oceanography.

[60] Roller BRK, Stoddard SF, Schmidt TM (2016). Exploiting rRNA operon copy number to investigate bacterial reproductive strategies. *Nature*. Microbiology.

[61] Stoddard SF, Smith BJ, Hein R, Roller BRK, Schmidt T (2015). M. rrnDB: improved tools for interpreting rRNA gene abundance in bacteria and archaea and a new foundation for future development. Nucleic Acids Research.

[62] LaRowe DE, Amend JP (2015). Catabolic rates, population sizes and doubling/replacement times of microorganisms in natural settings. American Journal of Science.

[63] Lomstein BA, Langerhuus AT, D’Hondt S, Jorgensen BB, Spivack AJ (2012). Endospore abundance, microbial growth and necromass turnover in deep sub-seafloor sediment. Nature.

[64] Vuillemin A (2019). Archaea dominate oxic subseafloor communities over multimillion-year time scales. Science Advances.

[65] Coskun ÖK, Özen V, Wankel SD, Orsi WD (2019). Quantifying population-specific growth in benthic bacterial communities under low oxygen using H218O. The ISME Journal.

[66] Eloe-Fadrosh EA, Ivanova NN, Woyke T, Kyrpides NC (2016). Metagenomics uncovers gaps in amplicon-based detection of microbial diversity. Nature Microbiology.

[67] Fischer MA, Güllert S, Neulinger SC, Streit WR, Schmitz RA (2016). Evaluation of 16S rRNA Gene Primer Pairs for Monitoring Microbial Community Structures Showed High Reproducibility within and Low Comparability between Datasets Generated with Multiple Archaeal and Bacterial Primer Pairs. Frontiers in Microbiology.

[68] Tully BJ, Heidelberg JF (2016). Potential mechanisms for microbial energy acquisition in oxic deep-sea sediments. Appl. Environ. Microbiol..

[69] Dekas AE, Poretsky RS, Orphan VJ (2009). Deep-sea Archaea fix and share nitrogen in methane-consuming microbial consortia. Science.

[70] Dekas AE, Chadwick GL, Bowles MW, Joye SB, Orphan VJ (2014). Spatial distribution of nitrogen fixation in methane seep sediment and the role of the ANME archaea. Environmental Microbiology.

[71] Joye SB (2004). The anaerobic oxidation of methane and sulfate reduction in sediments from Gulf of Mexico cold seeps. Chemical Geology.

[72] Barco RA (2015). New insight into microbial iron oxidation as revealed by the proteomic profile of an obligate iron-oxidizing chemolithoautotroph. Appl. Environ. Microbiol..

[73] Dyksma S, Pjevac P, Ovanesov K, Mussmann M (2018). Evidence for H2 consumption by uncultured Desulfobacterales in coastal sediments. Environmental microbiology.

[74] Castelle CJ (2013). Extraordinary phylogenetic diversity and metabolic versatility in aquifer sediment. Nature Communications.

[75] Meng J (2014). Genetic and functional properties of uncultivated MCG archaea assessed by metagenome and gene expression analyses. The ISME journal.

[76] Dahle H, Okland I, Thorseth IH, Pederesen RB, Steen IH (2015). Energy landscapes shape microbial communities in hydrothermal systems on the Arctic Mid-Ocean Ridge. ISME Journal.

[77] Mußmann M, Pjevac P, Kruger K, Dyksma S (2017). Genomic repertoire of the Woeseiaceae/JTB255, cosmopolitan and abundant core members of microbial communities in marine sediments. ISME Journal.

[78] Ettwig KF (2010). Nitrite-driven anaerobic methane oxidation by oxygenic bacteria. Nature.

[79] van de Vossenberg J (2013). The metagenome of the marine anammox bacterium ‘Candidatus Scalindua profunda’ illustrates the versatility of this globally important nitrogen cycle bacterium. Environmental Microbiology.

[80] Craine JM, Morrow C, Fierer N (2007). Microbial nitrogen limitation increases decomposition. Ecology.

[81] Musat F, Harder J, Widdel F (2006). Study of nitrogen fixation in microbial communities of oil-contaminated marine sediment microcosms. Environmental Microbiology.

