# Supplementary material for: Metagenomic views of microbial dynamics influenced by hydrocarbon seepage in sediments of the Gulf of Mexico
Source: Sci Rep. 2020 Apr 1;10:5772. doi: 10.1038/s41598-020-62840-z (PMC7113308; doi:10.1038/s41598-020-62840-z)
Supplement: Supplementary file 1 — Supplementary Information. [file 41598_2020_62840_MOESM1_ESM.docx]

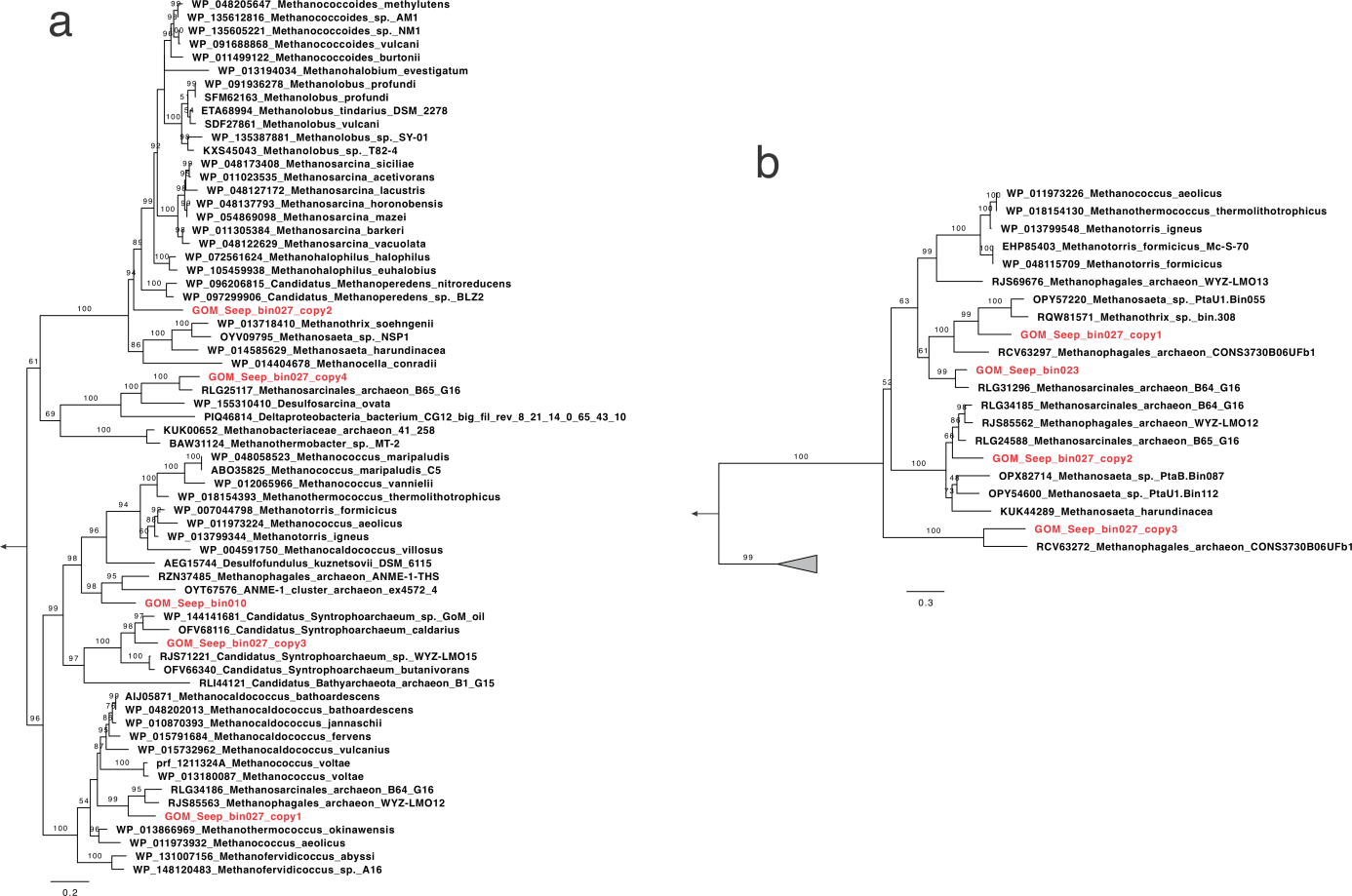


**Figure S1. Maximum-likelihood phylogenetic tree of NifH (a) and NifK (b) of Euryarchaeota.** The two trees were inferred using IQ-TREE v1.6.10 ^51^ with LG+I+G4 (a, NifH) and LG+G4 (b, NifK), respectively, as the best-fit evolutional model and 1000 ultrafast bootstraps. Both trees were rooted to the corresponding sequences of *Azotobacter vinelandii*. MAGs recovered from GOM in this study are highlighted in red. The scale bars show estimated sequence substitutions per residue.


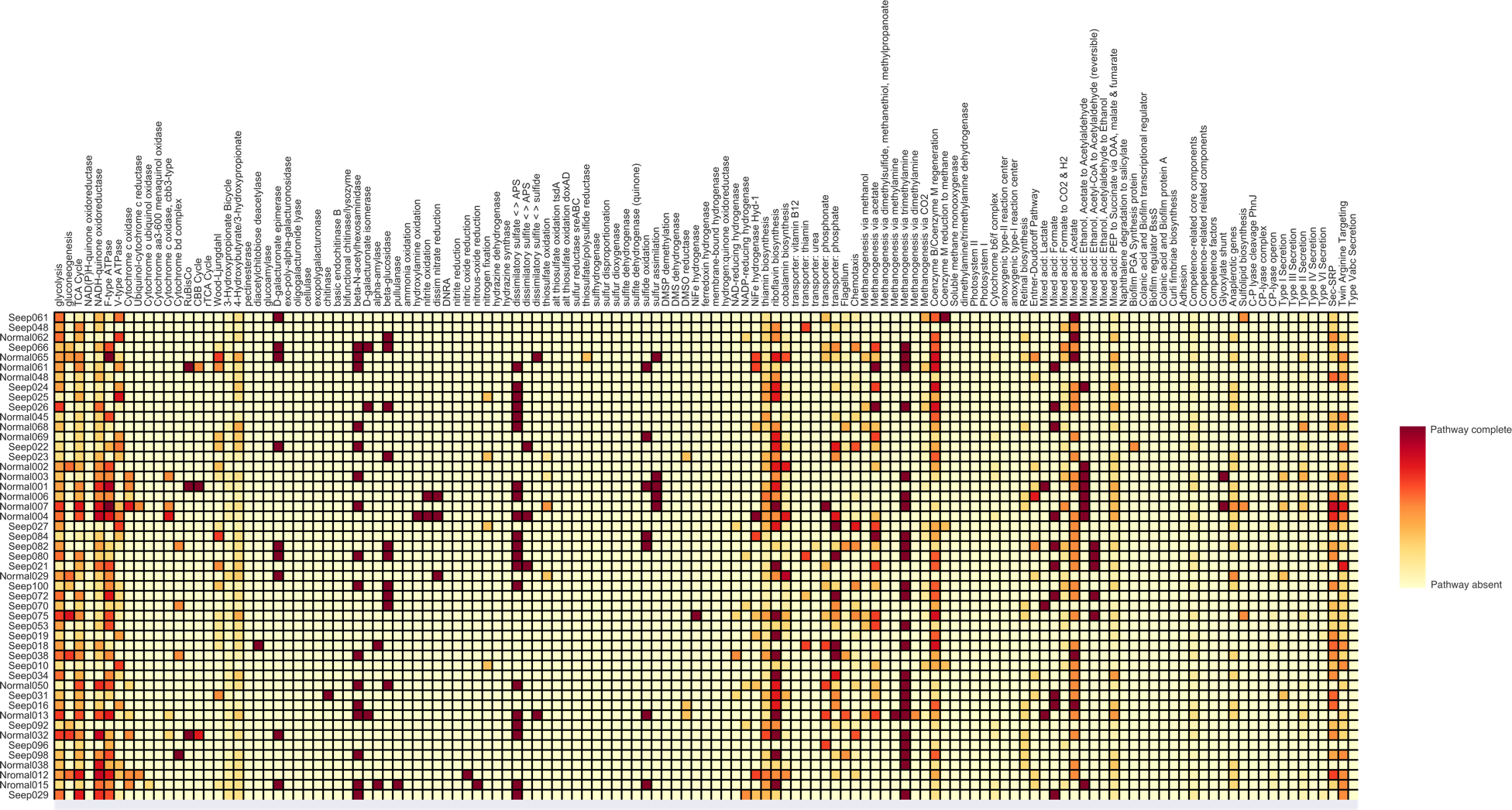


**Figure S2. Metabolic pathways encoded by the MAGs recovered from GOM sediments.** The overall metabolic capacities of MAGs were visualized through a heatmap prepared using KEGG-decoder V1.0.6-1.0.8 ^40^ based on the annotation results from KEGG annotation. Each square represents the occurrence of a pathway in a particular MAG. The darker the square is, the more completed the pathway is.
